# Advancing global health through engineering: a perspective on teaching an online global health course to engineers during a global pandemic

**DOI:** 10.1186/s12938-021-00910-7

**Published:** 2021-08-11

**Authors:** Seema Biswas, Oren Dahan, Evgeny Solomonov, Igor Waksman, Orit Braun Benyamin

**Affiliations:** 1 Global Health Research Laboratory, General Surgery B, Galilee Medical Center, Nahariya, Galilee Israel; 2 University of Bar Ilan, Azrieli Faculty of Medicine, Tzfat, Galilee Israel; 3 Mechanical Engineering, Ort Braude College, Karmiel, Galilee Israel

**Keywords:** Global Health, Engineering Education, Online learning, Assessment, Innovation

## Abstract

**Background:**

The effect of the COVID-19 pandemic on higher education has been felt worldwide. There are many lessons to be learned about teaching and learning in the digital age. While we evaluate the full impact and prepare ourselves for the new normal, it is worth reflecting on some of the positive aspects of online teaching and learning and understanding how students, teachers and the wider faculty have been able to support each other through the challenges of the pandemic. In this article, we offer a perspective on teaching an online Global Health course to engineering students.

**Results:**

The course, taught by a physician, provides a grounding in basic medical, scientific and engineering principles and is available to students of diverse engineering specialties. Students developed skills and gained confidence in active listening, sourcing and critical appraisal of information, interdisciplinary teamwork, needs assessment, problem analysis, problem-solving, effective communication, and organisation and delivery of information (in English). Students learned the importance of engineering in landmark historical public health projects, the delivery of modern health care, and the pressing need to develop engineering solutions to current global health problems. Course assessment was formative: 20% attendance and active participation in online classes, 30% problem-solving, 30% student presentations, and 20% written abstracts for two class projects: historical innovations and medicine in the future.

**Conclusions:**

We show how, through conversion from a classroom to an online format, we were able to deliver a rich curriculum with sound assessment where students were able to innovate together and discover the importance of engineering in health and well-being as we all experience an unprecedented global health pandemic.

## Background

Much has been written about the deleterious impact of the COVID-19 pandemic on higher education [1, 2]. The preparedness of institutions for large-scale digital learning has been a significant factor in overcoming challenges [1, 3], so, too, the conversion of classroom curricula to online formats [4, 5]. The latter is perhaps easier for smaller courses. We offer our perspective on teaching an online Global Health Engineering course during the pandemic.

### The course

In 2018, a new course, *Advancing Global Health Through Engineering*, was begun at Ort Braude College of Engineering in Israel. The course was originally designed for local and overseas students on the college International Study Abroad Program. Since the COVID-19 pandemic travel restrictions the course has become an online general course for local students. A physician with international medical experience, including work in low-resource settings, teaches the 13-week course in English.

The aims of the course are to provide engineering students an overview of how engineering impacts personal and public health, how engineering innovations may be used to promote healthy lifestyles (mitigating the effects of ill-health and disability) and to facilitate brainstorming amongst students with diverse engineering backgrounds (presenting these ideas to each other in English with confidence and enthusiasm). Students learn the importance of engineering in landmark historical public health projects, the delivery of modern health care, and the pressing need to develop engineering solutions to current global health problems. Some of these problems include global food shortages and healthcare inequalities, innovative solutions to community health problems such as home-monitoring of chronic diseases, and the importance of working closely with the community on sustainable and low-cost engineering programmes.

From the outset the intent of the course has been to promote enquiry about health needs and problem-solve engineering solutions in pairs in class. The course has evolved from a focus on local community needs and interaction with the local community to the latest course in 2020/2021 during the COVID-19 pandemic. During the pandemic all teaching and learning was conducted online in weekly Zoom sessions. Inevitably, students focused more on their own and their families’ health needs, including specific COVID-19 health solutions. Self-isolation and online teaching altered the way in which the course was taught and how students learned. The fundamental teaching and learning aspects of the course remained the same. These aspects included readiness and preparation for each class in advance, an emphasis on class participation and interaction with other students and the teacher, problem-solving during class, sourcing relevant information (usually online searches), and finally, presentation and abstract writing skills in English with the guidance of the teacher. Students were encouraged not to write out in full or read out what they wanted to present and not to rely on wordy PowerPoint slides in their presentations. Instead, they prepared slides with relevant images that served only as visual aids and practised speaking in pairs or with the teacher until they were ready to present fluently and hold the attention of their audience. The emphasis was on an explanation of the problem and exactly how their solution works. They were encouraged to discuss advantages and disadvantages, as well as how to test the function and efficiency of their solution.

Course materials, including the syllabus, weekly lectures—incorporating short videos about the topic, guidance on coursework and assignments, a breakdown of course assessment, and a reading list were uploaded prior to the start of the course on the college virtual online forum—the Moodle. As a result of occasional difficulties with online connections during the COVID-19 pandemic, lecture recordings were also emailed to students after each class. The slides, lecture recordings, suggested reading and videos served as preparation for each class. Crucial to each session was that the students had studied these and were ready for an interactive lesson.

In this article, we discuss some of the challenges and the benefits of going online with the teaching and learning of such a course. Through adaptations of the course syllabus and curriculum, and student feedback, we illustrate how learning objectives were achieved and how students adapted to online teaching and learning. We review more widely the goals of blending engineering and medical teaching and look at the potential for multidisciplinary collaborations in dealing with the world’s global health problems.

### Course structure

A 13-week course has been taught for four consecutive academic years. Each session comprises 2 h of “classroom” teaching every week. The course is taught by a physician with a background in global health and open to students of electrical, mechanical, software, biotechnology, and industrial engineering. Typically, 6–8 students join the course—2 of whom are non-Israeli international students. As all international students returned to their home nations at the outbreak of the COVID-19 pandemic, the current online course has only Israeli students.

The course was initially taught in person in class with an interactive 20-min lecture (with stated learning objectives, links to videos, further reading, and guidance on assignments). The lecture was followed by a class assignment designed to take the concepts introduced further. Students worked through solutions entirely original or based in-part on engineering solutions already in existence. After brainstorming in pairs or individually, students briefly presented their ideas using graphics or PowerPoint visual aids at the end of each 2-h session

With the conversion to a 100% online format during the pandemic, after the first two introductory sessions, from the third week onwards, sessions blended work from the previous week with new work. Each session began with a quick recap of the lecture of the previous week, student presentations of their engineering solution from the previous week individually or in pairs, a break of 5–10 min, a lecture on a new topic, and finally, brainstorming and problem-solving a solution based on this new topic. Thus, students had a week between sessions to finalize their weekly presentation in pairs. This gave them time to research, listen again to the lecture recording, and check their work with the teacher by email, Zoom, or WhatsApp. The recording of the relevant lecture served as a reminder of the topic and assignment, as well as a tool to reinforce-specific learning objectives.

Course structure on campus and online comprised three main components (Table 1). The aim of both campus and online teaching was to develop skills and build confidence in active listening, sourcing and critical appraisal of information, interdisciplinary teamwork, needs assessment, problem analysis, problem-solving, effective communication between peers, with health personnel and with the community, and organisation and delivery of information (in English). Students from different engineering backgrounds and different academic years were able to pair together to share knowledge and build skills in a layered approach towards formal presentations that revealed cumulative skills and were expertly delivered.

**Table 1:** Course structure: the three components of the course with examples of topics covered

Course components		Number of sessions	Topic examples
1	Address a community/family/individual health need	9	-Road safety -Disability -Safety in the home -Exercise at work -Food technology -Monitoring chronic disease -Oxygen delivery -Waste disposal -Sanitation -eHealthcare/telemedicine -Prevention of spread of COVID-19 and infection -Powering medical devices in low-resource settings
2	Research a historical engineering innovation that changed public health	2	-Clean water and sewage networks -Hospital waste management -Waste collection and recycling -Computer tomography (CT) -Magnetic resonance imaging (MRI) -Steam power -Clean energy sources -Electricity -Extracorporeal membrane oxygenation (ECMO)
3	Innovate for an unmet health need with technology of the future	2	-Paraplegia: exoskeleton, virtual reality, nerve/muscle stimulation -Oxygen extraction from the sea -GPS for the blind -Home ECG -Home prescription dispenser -Ultrasonic migraine detector -Gas leak sensor -CO_2_ absorption through biotechnology -P53 targeted chemotherapy -Anaesthesia through brain inhibition -Remote robotic surgery

Table 1 gives examples of individual lecture topics covered in the three components of the course: (1) addressing community health needs; (2) historical innovations that have changed public health; and (3) innovations for future healthcare. Learning domains for each teaching and learning method used on the course and specific learning objectives for each are listed in Table 2.

**Table 2:** Learning domains and specific learning objectives

Teaching and learning method	Learning domain	Learning objectives
Interactive lecture	-Key knowledge relating to medicine, science and basic engineering concepts -Grasp basic concepts in health and science -Evaluate the effectiveness of solutions -Listening and interacting with rest of class -Readiness to contribute and share ideas	-The effects of forces on the body in trauma -Principles of atherosclerosis causing heart disease, stroke, and hypertension -Pathophysiology of diabetes, asthma and epilepsy -Describe the effects of exercise on muscle and metabolism -Give a definition of global health -Give examples of engineering projects that impact the social determinants of health -Describe the properties of materials used in engineering -Define risk and hazards -Describe the principles of effectiveness and efficiency of machines
Listening to presentations from students	-Pay attention and actively participate in class -Listening and interacting with rest of class -Listen, offer suggestions, answer questions -Rate effectiveness of solutions -Readiness to contribute and share ideas	-Offer constructive feedback -Formulate questions -Construct reasoned arguments -Back up assertions with evidence
Research and problem-solving in class	-Organise information and classify importance -Apply basic engineering concepts to health problems -Analyse problems -Proficiency in critical appraisal of online information	-Formulate research questions -Source reliable information (in local languages and English) -Critically appraise information -Perform health needs assessment
Preparation and presentation	-Organise information and classify importance -Learning presentation skills -Gaining proficiency in preparing presentations and presenting -Gaining proficiency in English comprehension and writing -Explanation of complex engineering mechanisms and physical concepts -Classroom specific presentation skills -Online specific presentation skills	-Design short, effective PowerPoint presentations -Speak fluently, with expertise, agency and confidence -Answer questions precisely and with expertise -Engage with the audience
Writing abstract	-Organise information and classify importance	-Provide written background, define problem, describe solution succinctly -Correct English and grammar

### Assessment

Course assessment was formative rather than summative—integral to building knowledge, skills, and confidence, and for students to learn from each other as well as the course teacher. Weighting was applied to distinct aspects:

- Attendance and active participation in online classes 20%
- Problem-solving in class 30%
- Presentations in class 30%
- Written abstracts for historical innovations and medicine in the future 20%

Students were encouraged to work with each other and the teacher between classes to brainstorm and prepare presentations. As well as focusing on content, particular emphasis was placed on format, presentation skills, and English language in preparation of PowerPoint presentations and abstracts. Drafts for revision were exchanged by email between students and the teacher.

Marking focused on the clarity of the ideas presented, patient and systematic problem-solving, a sound understanding and explanation of the scientific basis of how each device works, and a class discussion of how the design might be improved.

**Fig. 1:**
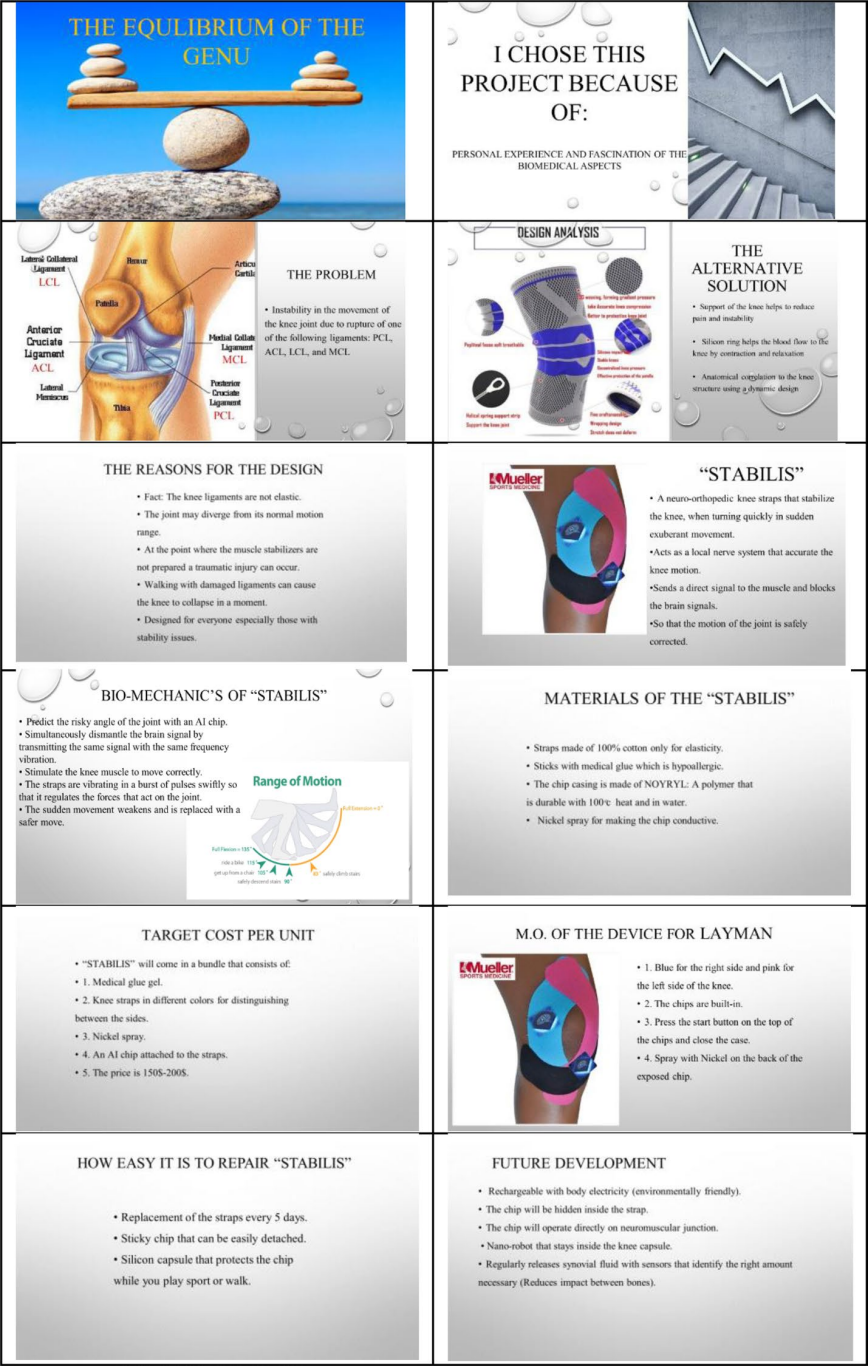
Example of student PowerPoint presentation of future innovations project

**Fig. 2:**
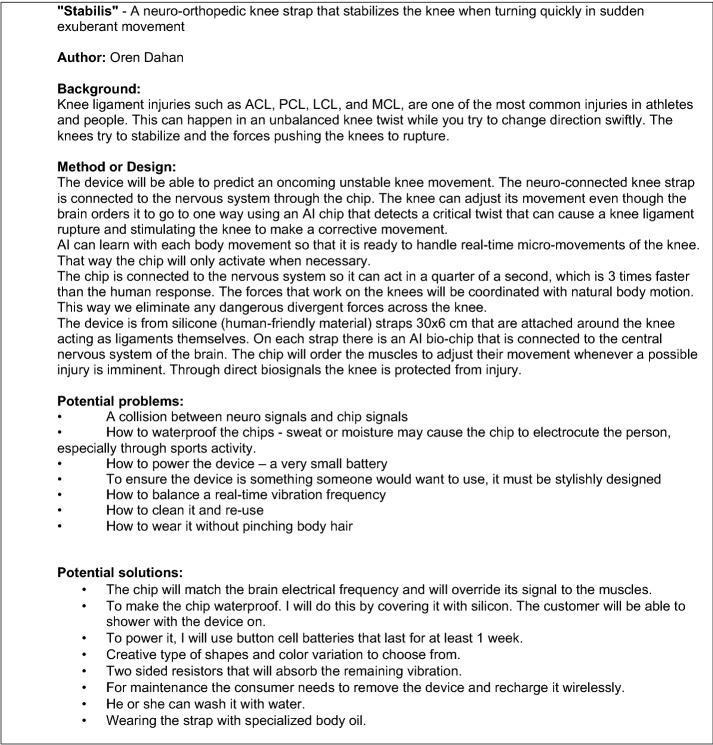
Example of student abstract (in style of conference submission)

Class participation included the formulation of incisive questions and the contribution to the debate in problem-solving and improvements of the design. Figures 1 and 2 show examples of the PowerPoint presentation and abstract of the future innovations project.

## Results

### Observations and outcomes

This course has always had a small number of students and always had its own WhatsApp group to supplement email and Moodle communication. Students offered verbal feedback (and ideas to mitigate problems with online connections, for example) throughout the course and consented to written feedback for use in improving the course, research and publication. This has been key to the high level of interaction of students with the teacher and with each other. There was a substantial investment of time in personal teaching and learning of engineering, health, science and language. The small class size is hugely advantageous in terms of building familiarity, fostering teamwork, a sense of togetherness, and overcoming initial nerves or shyness in interaction or presentation. Missed sessions were easy to make up online in student pairs. Student feedback indicates these as strong mitigators of the lack of face-to-face contact they miss while socially distancing or isolating.

### The three course components

#### Addressing a community health need

Individual sessions on specific community health needs comprised the majority of the course. The sessions highlight specific health needs and elaborate on the social determinants of health: access to clean water, to electricity, transport networks, safe home environments—all dependent on feats of engineering. Sessions were formulaic (online sessions beginning with student presentations of the previous week’s topic and ending with the lecture on the following week’s topic). The selection of weekly topics (Table 2) was based on feedback from students studying a wide range of engineering specialties. Topics were selected to appeal to specific engineering specialties, but also to have personal relevance to all students, and to fire the imagination. Thus, while telemedicine and disease monitoring applications (apps) suited the software engineers, they also inspired industrial and mechanical engineers. The most frequent problem students sought to address was a health-related issue of a family member or illness or injury they had suffered themselves—from disability aids for a grandparent to sports injuries of their own. The solutions were concepts that had to be based on workable principles. Although never created in prototype, they had to be practical and stand up to the scrutiny of peers and, of course, the teacher. Solutions ranged from simple, practical concepts (stands designed for computer tablets for stroke patients, for example) to the inspired (pressurised oxygen delivery to deep sea divers that might prevent divers bends) again, examples of problems students had encountered personally.

Both classroom and online classes were well-attended. An advantage of the togetherness of students was that students always came prepared to class, were ready to share their ideas, offer suggestions to each other and encouraged each other to switch on their cameras on Zoom. Indeed, both verbal and written student feedback on the course insisted that cameras should be switched on where possible.

While all students said they would have preferred a face-to-face class on campus, they cited presenting online as less intimidating than standing in front of a class, enjoyed the reverse classroom format that enriched what they gained from each session, felt better able to take notes and look up information online during class, and enjoyed working in pairs online in breakout groups—undisturbed and quieter than a classroom. They also cited the advantages of not travelling to class, and while some online courses afforded them a certain amount of privacy and flexibility in participation, they cited the high level of engagement and participation necessary of this course as an advantage and motivator.

While at least half of the students knew little or nothing of the course before they enrolled, by the third week, it became obvious that to gain from the course, attendance and active participation were obligatory, and there was nothing to gain from attendance without preparation beforehand as the discussion had moved well beyond that of the previous week. Thus, to keep up and thrive, students needed to prepare well. To reduce the amount to commitment this required at home, time in class was dedicated to preparation of assignments in breakout groups, with the teacher on-hand to assist.

One interesting feature of the class was that the students always chose their own partners with whom to work (on campus and online). Occasionally, students worked alone but their choice of partner was almost always from a different engineering background, a different academic year and, when international students were present, they always paired with an Israeli.

Although discussion between the student pairs was sometimes in Hebrew or Arabic, discussion between groups, with the teacher and with international students was always in English. Illustrations, graphics, presentations and written work were always in English. Students were expected to question each other in English after each presentation and were made aware that the quality of the questions and the answers was assessed. The standard expected was of complete fluency in English, thus, all students were anxious to improve their English and hand in assignments that were proofread by the teacher. Weekly presentations of smaller class assignments were designed to hone skills needed for the two larger assignments. In reality, only minimal editing was required of the teacher.

Two striking observations of the online format of the course were that students forced up the standard of their work. With consecutive classes, students were heavily influenced by the amount of effort their peers would invest in their research and presentations. They would remark on this in class feedback, but, more importantly, drive up the standard of their own work the following week. The second, and related observation was that the amount of research students undertook increased with each week, seemingly seeking to outdo their performance in their last assignment and answer questions from their peers as experts. Indeed, the students substantially exceeded the expectations and requirements of the course.

#### Historical innovations

The study of historical innovations in engineering was originally designed to foster research, and skills in presentation and abstract writing. In the campus-based course an initial lecture outlined how historic engineering feats had changed humanity. Examples discussed were the design and construction of the Paris sewer system and modern surgical operating theatres. Students then studied these and similar examples and focused on the engineering challenges of that era and how these were overcome. They presented their thoughts to the class. Already a favourite of the students, however, online, this assignment leapt forward. Again, based on the experiences of family and loved ones, students wanted to study aspects of cancer treatment, radiological imaging, surgical equipment, and set about detailed research of the development of gene therapy, computer tomography (CT), magnetic resonance imaging (MRI) and extracorporeal membrane oxygenation (ECMO). Other projects, also inspired by personal history, described the development of electricity and the first electrical power supplies to their hometowns in Galilee. Students chose their own topics, brainstormed in breakout groups and continued their research at home. Sharing their work with the teacher, they structured their presentations and prepared written abstracts.

The standard (and time-keeping requirements) of presentations followed typical conference guidelines—intended to be practice for national and international conferences. Written abstracts similarly followed usual conference requirements. Detailed guidance and examples were provided to students. Rough drafts were improved upon in class and at home with guidance from the teacher. The abstracts comprised the two written assignments of the course: Historical Innovations and Future Healthcare. Abstracts were structured, had word limits and deadlines (as per a typical conference) and were emailed to the rest of the class in advance of final presentations. Students were expected to read these and formulate questions to ask after listening to each presentation.

#### Future healthcare

If the study of historical innovations was a favourite assignment of the students, according to their feedback and endeavours, innovating future technology to cure disease or improve health was perhaps the most inspiring part of the course. The introductory lecture focused on one topic as an example with three solutions that are the subject of research and development at present. The example of paraplegia resulting from trauma was used with news and documentary footage of three technological innovations—virtual reality, spinal implants and robotic exoskeletons. Using these as examples, students chose their own topics to research. Although the scientific possibilities required solid grounding, the opportunity to imagine the ‘impossible’ was an inspiration. Students researched deep brain stimulation and neuronal inhibition to anesthetise patients using electrical signals rather than drugs, corrected joint abnormalities by stimulating weak, denervated muscles externally through a carefully designed knee brace to detect and correct abnormal movement in a fraction of a second. Software engineering students paired with biotechnology students explained to the class the role of oncogenes and targeted gene therapy for cancer.

From a teaching perspective, this is perhaps the highlight of the course. Perhaps because this is the final assignment of the course, or perhaps because the students are well-prepared having completed the rest of the course, this assignment requires no encouragement, no reminder and only pointed medical advice. The students are ready, enthusiastic, and their ideas far-fetched yet practicable solutions to problems that, at first, might seem beyond our reach to take on. In a pandemic with bad news more often than good, students debate technology that might just be within our grasp. Certainly, these are the students to shape global health in the future.

## Conclusion

Crucial to the success of the course was to include topics that all students might engage with. These topics were based on student suggestions and reflected more broadly the particular disciplines students were studying for their undergraduate engineering degrees (from food technology to disability aids, for example). The topics, also, of course, reflect the scope of engineering across all walks of life, and are a reminder to us of the importance of basic science, simple mechanics and a familiarity with technology necessary in modern life.

Active class participation, the incorporation of feedback and good-natured competition were drivers for learning and improved standards. The projects on historical and future engineering innovations for health fired the imagination and have, since the course, been incorporated by students into other projects toward their engineering degree.

Biomedical and biotechnological engineering courses are becoming a strong feature of the global engineering curriculum [6–8]. Designed for engineers who might work in medical laboratories, engineer medical software and develop materials that change the way people live with disability, there is much to learn. The volume of biomedical science alone is overwhelming. Exponential advances in biomedical technology add to the teaching and learning challenges of a rapidly evolving field. Yet, there is a need for a course that brings together medical problems and their engineering solutions for engineers of all backgrounds. Engineers of all specialties impact the way in which people live with disease and disability. Historical engineering innovations have changed public health. Future innovations will change global health. Courses that provide a grounding in basic medical, scientific and engineering principles have a role to play in the education of all engineers. Online education is no bar to a good education with sound assessment.

The COVID-19 pandemic had brought home the need for the medical and engineering sectors to work together. We need to understand each other and the needs of the people we aim to help if we are to be effective. We need, as this course begins and ends, to “look about us and see that everything we live with, depend on, and often overlook has been engineered”.

## Data Availability

Supplementary data are available from the authors on request.

## References

[1] Giorgio M, Van’t Land H, Jensen T. The impact of COVID-19 on higher education around the World IAU Global Survey report. 2020. https://www.iau-aiu.net/IAU-Global-Survey-on-the-Impact-of-COVID-19-on-Higher-Education-around-the. Accessed 27 July 2021.

[2] Aristovnik A, Keržič D, Ravšelj D, Tomaževič N, Umek L (2020). Impacts of the COVID-19 pandemic on life of higher education students: a global perspective. Sustainability.

[3] Agasisti T, Soncin M (2021). Higher education in troubled times: on the impact of COVID-19 in Italy. Stud High Educ.

[4] Kebritchi M, Lipschuetz A, Santiague L (2017). Issues and challenges for teaching successful online courses in higher education: a literature review. J Educ Technol Syst.

[5] Mishra L, Gupta T, Shree A (2020). Online teaching-learning in higher education during lockdown period of COVID-19 pandemic. Int J Educ Res Open.

[6] Chien S, Bashir R, Nerem RM, Pettigrew R (2015). Engineering as a new frontier for translational medicine. Sci Transl Med.

[7] Rambukwella M, Balamurugan A, Klapholz H (2021). The application of engineering principles and practices to medical education: preparing the next generation of physicians. Med Sci Educ.

[8] Kurpinski K, Johnson T, Kumar S, Desai T, Li S (2014). Mastering translational medicine: interdisciplinary education for a new generation. Sci Transl Med.

